# Are Overweight and Obesity Risk Factors for Developing Metabolic Syndrome or Hypertension after a Preeclamptic Event?

**DOI:** 10.3390/healthcare11212872

**Published:** 2023-10-31

**Authors:** Maria Luisa Pizano-Zarate, Yessica Dorin Torres-Ramos, Rosa Maria Morales-Hernandez, Maria Cristina Ramirez-Gonzalez, Maria Hernandez-Trejo

**Affiliations:** 1 Department of Nutrition and Bio-Programming, Instituto Nacional de Perinatologia, Montes Urales 800, Ciudad de Mexico 11000, Mexico; 2 Immunobiochemistry Department, Instituto Nacional de Perinatologia, Montes Urales 800, Ciudad de Mexico 11000, Mexico; 3 Department of Developmental Neurobiology, Instituto Nacional de Perinatologia, Montes Urales 800, Ciudad de Mexico 11000, Mexico

**Keywords:** preeclampsia, overweight, obesity, hypertension, metabolic syndrome, adiposity post-childbirth

## Abstract

Objective: To identify the determinants and risks associated with developing hypertension and metabolic syndrome in the first year postpartum in women who experienced preeclampsia. Methods: A cohort study was conducted, involving women who had experienced preeclampsia (PE) recently. The control group was women with the same characteristics but a healthy pregnancy. The variables analyzed were somatometry, disease history, pre-pregnancy body mass index (Pre-BMI), and Third Adult Treatment Panel updated (ATP III) metabolic syndrome (MS) data (blood pressure, obesity, triglycerides, high-density lipoproteins, and fasting glucose). These variables were measured at 3, 6, and 12 months postpartum. Results: Women with a history of PE exhibited higher systolic and diastolic blood pressure than women without PE. The risk of developing isolated diastolic arterial hypertension at 3 and 12 months of follow-up was two to eight times greater in women with a history of PE. Factors associated with having higher blood pressure levels were preeclampsia, insulin resistance, age, and BMI. Neither the pre-BMI index nor gestational weight gain (GWG) had any effect on blood pressure in any of the three assessments. Women with preeclampsia had a 5- to 8-fold increased risk of developing MS (which could be explained not only by the history of preeclampsia but also by the history of pre-pregnancy obesity). However, PE was not identified as a risk factor at the six-month evaluation and was only explained by pre-pregnancy obesity and overweight. Conclusions: Obesity and overweight, as well as preeclampsia, were strongly associated with the development of hypertension and metabolic syndrome during the first year following childbirth.

## 1. Introduction

Preeclampsia (PE) is a hypertensive disorder presenting after the 20th week of pregnancy, may or may not be accompanied by proteinuria and a low platelet count, with the involvement of other organs, and affects 2–8% of first pregnancies [1,2,3,4]. In recent years, the international literature has presented interesting advances in understanding the pathophysiological processes that induce hypertensive disease during pregnancy, including preeclampsia. We now recognize two forms of presentation of preeclampsia: late-onset preeclampsia, which typically occurs after the 34th week of gestation and is associated with maternal vascular predispositions, good perinatal outcomes, but higher maternal mortality and morbidity, and a linear correlation with increased maternal Body Mass Index (BMI); and early-onset preeclampsia (EOP), which occurs before the 34th week and is believed to have an immunological etiology, strongly associated with primiparity. EOP poses serious risks to both the mother and fetus. Low-income countries report EOP in over 30% of preeclampsia cases, whereas countries with higher economic resources (accounting for 12% of global births) report only 10% of EOP cases [5,6]. Multiple studies indicate an increased risk of cardiovascular disease, including ischemic heart disease and cardiovascular death, and a fourfold increased risk of stroke in later years following a hypertensive disorder during pregnancy [7,8,9].

Conversely, prior to pregnancy, women with metabolic syndrome (MS) may face a higher risk of developing preeclampsia and diabetes during pregnancy [10,11]. Additionally, evidence suggests that women who experience preeclampsia during pregnancy are more likely to develop MS and diabetes postpartum or later [11,12]. Maternal risk factors for placental syndromes include obesity, insulin resistance, dyslipidemia, and chronic high blood pressure. All of them are also independent predictors of adult-onset cardiovascular disease [13]. Maternal placental syndromes are complications that occur due to placentation disorders, that is, during the creation of the placenta. That usually alters its functioning. Preeclampsia, gestational hypertension, intrauterine growth restriction, pregnancy loss, and stillbirth often have abnormal placentation as their etiopathogenesis [14]. Although blood pressure levels in preeclampsia usually normalize after childbirth, there is a risk of persistent systemic arterial hypertension.

This study aimed to identify the determinants and risks associated with developing hypertension and metabolic syndrome in the first year after childbirth in women with a recent history of preeclampsia, compared to women without this antecedent (controls).

## 2. Methods

### 2.1. Study Population

To recruit patients, we consulted the medical records of women who gave birth within the last 45 days, using a consecutive approach. This non-probabilistic study was conducted at the National Institute of Perinatology in Mexico City, a tertiary government institution that caters to the uninsured population (from October 2014 to April 2017) in a cohort of women between 18 and 45 years of age with preeclampsia in the last pregnancy. All patients had to comply with the selection criteria (women who gave birth to a single child, were not currently pregnant, had no history of pre-existing carbohydrate intolerance, metabolic syndrome, or diabetes mellitus, and freely consented to participate). Additionally, participants should not have taken medications during and after the pregnancy that interfere with the variables of interest (like hypoglycemic agents, antihypertensive agents, glucocorticoids, lipid-lowering agents, thyroid hormones, and hormonal contraceptives). Elimination criteria were: women who were detected—during the follow-up—with pathologies such as hyperthyroidism, hypothyroidism, kidney damage, liver failure, or who became pregnant again.

The controls were recruited consecutively, at the same time as the cases, matching by age and by the (aforementioned) inclusion and exclusion criteria, among patients diagnosed with normal-evolutionary pregnancy (without pathologies). All eligible participants still hospitalized were personally invited to participate, while those who had been discharged were contacted by telephone. A total of 261 participants met the research criteria and provided informed consent. Among them, 148 had preeclampsia (52 severe and 96 mild), while 113 controls without preeclampsia were recruited.

### 2.2. Clinical and Demographic Data

The diagnosis of preeclampsia was made during pregnancy by senior obstetrics and gynecology specialists in the clinic, commonly based on the criteria of the National High Blood Pressure Education Program Working Group on High Blood Pressure in Pregnancy [15]. The FIGO Initiative defines preeclampsia as “gestational hypertension” accompanied by ≥1 of the following new-onset conditions at or after 20 weeks of gestation:

1. Proteinuria (i.e., ≥30 mg/mol protein:creatinine ratio; ≥300 mg/24 h; or ≥2+ dipstick).
2. Other maternal organ dysfunctions include:

Acute kidney injury (creatinine ≥90 μmol/L; 1 mg/dL), liver involvement (elevated transaminases, e.g., alanine aminotransferase or aspartate aminotransferase >40 IU/L) with or without right upper quadrant or epigastric abdominal pain, neurological complications (e.g., eclampsia, altered mental status, blindness, stroke, clonus, severe headaches, and persistent visual scotomata), or hematological complications (thrombocytopenia-platelet count <150,000/μL, disseminated intravascular coagulation, hemolysis).

3. Uteroplacental dysfunction (such as fetal growth restriction, abnormal umbilical artery [UA] Doppler waveform analysis, or stillbirth) [2].

Although the International Society for the Study of Hypertension in Pregnancy (ISSHP) now does not recommend classifying preeclampsia as moderate or severe [16], at the time this work was carried out, preeclampsia in the clinical field was defined as moderate when the systolic blood pressure figures were ≥140 mm Hg and diastolic ≥ 90 mm Hg (in the doctor’s office) and severe preeclampsia if the blood pressure systolic pressure was >160 mm Hg and/or >110 diastolic pressure.

General demographic characteristics, pathological and obstetric history, as well as pre-pregnancy weight and height, were collected. Pre-pregnancy BMI [17] and gestational weight gain were then calculated.

The research adhered to the principles of the Declaration of Helsinki, always ensuring the confidentiality of the study subjects. It received approval from the ethics, research, and biological sample handling committees (Protocol code 3210-21001-02-14 on 12 December 2019).

The variables analyzed were somatometry and clinical characteristics of ATP III metabolic syndrome (MS) (blood pressure, obesity, triglycerides, high-density lipoproteins, and fasting glucose). These variables were measured at 3, 6, and 12 months postpartum.

### 2.3. Participants’ Follow-Up

The participants’ follow-up was conducted with three evaluations at 3, 6, and 12 months after the birth of their children. Each evaluation consisted of the following:

(a) Somatometry: This included measurements of weight, height, waist circumference, and hip circumference. Body weight was obtained under normal conditions using a TANITA digital scale (Tokyo, Japan, Model BMB 800). Height was assessed using a SECA stadiometer (Hamburg, Germany, Model 208). Anthropometric measurements were taken following the Centers for Disease Control and Prevention guidelines (CDC) [18].
(b) Blood pressure. Blood pressure measurements were performed using the auscultatory method in triplicate, simultaneously by a doctor and a nurse, both standardized with the Habicht method [19].
(c) Blood pressure was determined after at least 10 min of rest using a sphygmomanometer and a cuff appropriate for the participant’s complexion. The measurements were taken in the morning by triplication with an interval of 2 to 3 min; the participant was seated, leaning on the chair’s backrest, with feet on the floor, and extended left arm. Simultaneous determinations were made by the doctor and nurse using a stethoscope with a double earpiece and a single capsule, allowing them to listen to the same patient at the same time. Systolic and diastolic blood pressure (SBP and DBP) were recorded, utilizing Korotkoff phases I and V as references, respectively. If there was a difference of more than 2 mm/Hg, the procedure was repeated after a 5-min rest. The final value was obtained by averaging the latter two readings. The cut-off points for diagnosing arterial hypertension in this study were based on the criteria established by the American College of Obstetrics and Gynecology, the European Society of Cardiology of 2018 criteria, and the WHO [20,21,22,23].
(d) Each time, participants were questioned about their medical history, including gynecology, obstetrics, and pathology.
(e) Blood samples were taken by venipuncture to determine glucose, cholesterol, triglycerides, and HDL quantities in serum or plasma. Quantitative in vitro determinations were performed using photometric equipment from System Kits DiaSys, Diagnostic Systems GmbH (Holzheim, Germany). Insulin levels were measured by ELISA using the chemiluminescence detection method. Equipment from the Beckman Coulter brand Access (Pasadena, California, CA, USA) was used. Insulin resistance was calculated by homeostasis model assessment (HOMA-IR). HOMA-IR = fasting glucose levels [mmol/L] × fasting insulin levels [μU/mL]/22.5.
(f) Somatometry of newborns was performed within the first 12 hours of life. A calibrated scale (SECA 374) and an infantometer (SECA 416) were used. Weight was classified according to the intergrowth criteria as small for gestational age (SGA) (percentile <10), adequate for gestational age (AGA) (10th to 90th percentile), and large for gestational age (LGA) (>90th percentile) [24].
(g) Metabolic syndrome (MS) was defined according to the updated Third Adult Treatment Panel (ATP III) version (criteria for adults): BMI ≥ 30 cm, along with any two of the following: raised triglycerides (≥1.69 mmol/L [150 mg/dL]), reduced high-density lipoprotein cholesterol (<1.29 mmol/L [<50 mg/dL]), raised blood pressure (BP) (i.e., systolic BP ≥ 130 mm Hg or diastolic BP ≥ 85 mm Hg), or raised plasma glucose (≥5.5 mmol/L). Since almost all women experience changes in waist circumference due to a larger gap between the rectus abdominal muscles, especially in the first year after delivery, the WHO and ATP III criteria were modified using a BMI equal to or greater than 30 kg/m^2^, which is why it is used instead of waist circumference [25,26].

### 2.4. Statistical Analysis

Our outcome variables were blood hypertension and metabolic syndrome at 3, 6, and 12 months post-childbirth. We performed a Pearson X^2^ to compare groups. As a risk, we calculated the odds ratio and prevalence ratios. We employed probabilistic models, treating PE as the main exposure variable. A univariate general linear model was performed to analyze the association of some maternal factors and covariates (from pre-pregnancy, pregnancy, and the first year postpartum) in order to adjust for possible confounding factors and find the exposure variables that could explain the main outcomes (blood hypertension and MS) at 3, 6, and 12 months post-childbirth. To identify factors influencing arterial hypertension after pregnancy in participants with severe, mild, or without preeclampsia, a general linear model was used. Independent variables included age, pre-pregnancy body mass index, weight gain during pregnancy, body mass index, homeostatic model assessment (HOMA), and history of previous preeclampsia at each evaluation. A logistic regression model was used to calculate the factors that could explain MS after pregnancy. The statistical analyses were performed using the standard statistical software package SPSS 25.0 (IBM SPSS Statistics).

## 3. Results

### 3.1. Risk of Preeclampsia

In our sample, 74 participants (28.3%) were primiparous, of whom 51 (69%) suffered from PE. Among 187 multiparous women, 97 (52%) had PE (*p* = 0.012). Compared with multiparous women, the risk of PE in primiparous women was determined to be 2.05 (95% CI 1.16–3.63).

Furthermore, among multiparous women, the risk of PE was 14.6 times higher if they had a previous personal history of PE (OR 14.6; 95% CI 6.5–32.5). Similarly, this risk was higher for mild PE (OR 17.6; 95% CI 7.5–41.2) compared to severe PE (OR 9.64; 95% CI 3.5–26.2), both of them compared with the control group that only had nine participants with a history of previous preeclampsia (all of them multiparous).

### 3.2. Analysis of Lost Participants

The initial sample consisted of 261 participants. Among them, 38 cases were lost between the first and second evaluations (3 severe PE, 16 mild PE, and 19 controls), and 29 cases were lost between the second and third evaluations (7 severe PE, 12 mild PE, and 10 controls) (Table 1).

In the 67 cases lost, no significant differences were found in age, pre-gestational body mass index, gestational age (*p* = 0.74), gestational weight gain (*p* = 0.70), PE severity grade, personal history of hypertension, or gestational diabetes between participants with and without full follow-up.

### 3.3. Baseline Characteristics of the Cohort

Table 2 presents a comparison of the main clinical characteristics between groups. The following findings are noteworthy:

1. Controls, who did not have preeclampsia, were predominantly thin (BMI < 24.9 kg/m^2^), while the more significant number of obese or overweight women had PE.
2. Out of all the participants, 36 had a personal history of arterial hypertension. Among them, 24 (66.7%) had mild PE, and 9 (25%) had severe PE. Only 3 of the 113 controls had a history of high blood pressure. Throughout the three evaluations, the blood pressure values of the patients in both groups (with and without preeclampsia) were similar between those with a personal history of arterial hypertension and those without.
3. Women with PE had a higher incidence of premature births (71%), and 87.5% of preeclamptic women had children with low birth weights.
4. Age, gestational diabetes, and pre-pregnancy diabetes mellitus did not differ between groups at the beginning of the study. All our cases of preeclampsia were late-onset.
5. Sixty-nine participants had a personal history of previous preeclampsia; nine (13%) belonged to the control group, and the vast majority (65.2%) belonged to the mild preeclampsia group.

### 3.4. Comparative Evolution of Systemic Blood Pressure after Pregnancy with or without Preeclampsia

When considering the cutoff points for arterial hypertension (systolic hypertension ≥ 140 mm Hg and diastolic hypertension ≥ 90 mm Hg) [18,19,20,21], it was found that three months after delivery, women with a history of PE had eight times more risk of having diastolic arterial hypertension (OR 8.17, CI 95% 1.86–35.8), but without high systolic blood pressure figures (isolated diastolic hypertension (IDH)) compared to women without PE. At the 6-month evaluation, none of the participants had high blood pressure readings. However, in the 12-month follow-up, isolated high diastolic arterial hypertension reoccurred, with an 83% higher risk of diastolic hypertension among women with PE in the previous pregnancy (Table 3).

IDH was the predominant phenotype of hypertension found in this sample. At three months postpartum, IDH was present in 92.8% of women with a history of PE (13 out of 14 women), while only one out of 14 women without PE (7.2%) had IDH. At six months postpartum, twelve women had IDH, with nine women (75%) being from the preeclampsia group and three (25%) from the control group. Finally, after one year, all seven women with IDH had a history of preeclampsia in their last pregnancy (100%).

According to the general linear model, it seems that the history of PE, whether it was severe or mild, cannot fully explain the systolic and diastolic blood pressure figures up to 12 months after pregnancy. According to Table 4, maternal age, body mass index, insulin resistance, and preeclampsia are factors that consistently explain the blood pressure values across all three assessments. Interestingly, the history of previous preeclampsia, pre-pregnancy BMI index, and gestational weight gain (GWG) did not have any association with blood pressure in any of the three assessments.

### 3.5. Metabolic Syndrome

Our study focused on using the recent ATP III criteria for diagnosing MS and examining the link between PE and the further development of MS. Our analysis of risks revealed inconsistent findings, with a significantly higher risk of MS observed in women who had experienced PE in their last pregnancy during the 3-month and 12-month post-childbirth evaluations. The odds ratios were 5.2 (95% CI 1.9–14) and 8.2 (95% CI 1.0–65), respectively. However, no association was observed between PE and MS during the 6-month evaluation, with an odds ratio of 1.6 (95% CI 0.6–4.1).

Additionally, we employed a logistic regression model to identify the factors that could explain the development of MS after pregnancy with PE. Maternal age, pre-pregnancy BMI, gestational weight gain, exclusive breastfeeding, a history of gestational diabetes, a history of previous preeclampsia, and blood levels of LDL-C were used as exposure variables.

At three months postpartum, obesity, exclusive breastfeeding, and preeclampsia (severe and moderate) were the variables that explained the MS (R^2^ 0.410). However, in the 6-month evaluation, only pregestational overweight and obesity explained the MS (R^2^ 0.295). Finally, at one year of follow-up, the MS was only associated with obesity but not with a history of preeclampsia (R^2^ 0.272) (Table 5).

### 3.6. Maternal Postpartum Adiposity

We sought to determine whether overweight and obesity, in addition to preeclampsia, played a significant role in the development of hypertension and metabolic syndrome in the first year postpartum. We also investigated the factors that could explain overweight and obesity in these women.

The body mass index one year postpartum was similar between women with PE and without PE (26.4 ± 4.1 vs. 25.5 ± 4.2, *p* = 0.13). In contrast, at 3 and 6 months of follow-up, the mean BMI between these two groups differed with statistical significance (27.6 ± 4.4 vs. 26.4 ± 4.1, *p* = 0.02, and 27.4 ± 4.3 vs. 25.8 ± 4.2, *p* = 0.005, respectively). Then we applied an analysis with a general linear model adjusted for age, breastfeeding practice, use of hormonal contraceptives, gestational weight gain, pre-pregnancy BMI, and having or not having PE during the last gestation. Table 6 describes that the variability of BMI in each of the three follow-up moments is explained by 65% to 73% (R^2^ adjusted) with only two variables: the pre-pregnancy body mass index and the gestational weight gain. The Waist Height Index variability was dependent on the pregestational BMI and the GWG, between 52% and 65%, in the three scrutinies. Interestingly, breastfeeding is added as a predictor factor at 12 months postpartum. Finally, the waist hip index at 6 and 12 months postpartum is barely explained, between 13% and 19%, by the same variables, adding the covariate age.

It is worth noting that preeclampsia had no significant effect on adiposity during the participants’ one-year follow-up period. Table 6 demonstrates that preeclampsia explained only 0% to 2% of the variability in the model.

Table 6 also clearly shows that the pre-BMI explains the postpartum variability of BMI in a percentage that oscillates between 63% and 71%.

## 4. Discussion

### 4.1. Preeclampsia and Subsequent Morbidity in the Mother

Between-group analysis of blood pressure at the three assessments of our participants (at 3, 6, and 12 months postpartum) showed that those who had a pregnancy with preeclampsia had significantly higher mean systolic and diastolic blood pressure values than controls. In the international literature, evidence of remodeling and structural and functional alterations of the heart has been observed in pregnancies with preeclampsia [27].

Moreover, strong evidence, including a meta-analysis of >2.3 million women, indicates that preeclampsia is associated with an increased risk of future cardiovascular disease. The relative risk for developing cardiovascular disease, including cerebrovascular disease, peripheral arterial disease, and cardiovascular mortality, was found to be 2.00 for mild preeclampsia, 2.99 for moderate preeclampsia, and 5.36 for severe preeclampsia [28]. They also revealed a graded relationship between the severity of preeclampsia and the risk of heart disease. Unfortunately, the majority of studies on the association between HDP and CVD focus on long-term CVD risk, and limited data are available to address early and midterm risks. Moreover, Veerbeek reported that 42% of women with preeclampsia and 39% of women with gestational hypertension progress to hypertension after a mean follow-up of 2.5 years, compared with rates as low as 1% among women with normotensive pregnancies [29]. On the other hand, Lykke found that the hypertension risk increased 3.6 times after mild preeclampsia and 6.0 times after severe PE [30].

### 4.2. High Recurrence of Preeclampsia

The literature shows that the recognized risk of PE among primiparous women is between 1.4 and 5.5 times higher than in multiparous women [29,30], as occurred among our patients. Some retrospective studies have found between 25 and 29% recurrence of preeclampsia [31,32,33,34].

In relation to the above, our case material showed a high number of cases of recurrence of preeclampsia (women with more than one pregnancy). The frequency of presentation of PE among multiparous women with a previous pregnancy with PE was 87% (60/69), while preeclampsia among multiparous women without a history of PE was 13.0% (9/69), (*p* < 0.001). A possible explanation for this high recurrence may be that the institution that makes this report specializes in high-risk pregnancies and is a national reference hospital; therefore, the cases are concentrated.

### 4.3. Adherence to the Follow-Up

Long-term follow-ups usually experience losses, as observed in the present study. Between the first and second evaluations, 14.5% of the participants were lost, and for the third screening, 11% were lost. However, the losses in this study were not statistically different from those who completed the follow-up, in terms of both exposure and outcome.

### 4.4. Chronic Hypertension and Preeclampsia Superimposed

Chronic hypertension in pregnant women is associated with an increased risk of developing preeclampsia, which further contributes to maternal and perinatal complications [35]. It has been reported that between 10 and 40% of women with chronic arterial hypertension develop preeclampsia, which leads to more maternal complications and poor perinatal outcomes [36]. In the current sample, 33 women had superimposed preeclampsia, with three cases (27%) classified as severe preeclampsia and 24 (73%) classified as moderate preeclampsia. However, the statistical model applied did not demonstrate that a history of chronic hypertension affected the blood pressure figures in any of the three scrutinies post-childbirth. The strict prenatal control that is usually performed on pregnant women with preeclampsia in our tertiary institution may have been one of the reasons for the good results of our cases with superimposed preeclampsia, at least during the first year after the event.

### 4.5. Risk of Arterial Hypertension

Throughout the year of follow-up, elevated diastolic blood pressure figures were found to be the most prevalent among the patients in this study. This aligns with findings from the US, where it is known that more than 45% of untreated hypertensive patients under the age of 50 have isolated diastolic hypertension (IDH). Being overweight or obese is strongly associated with this hypertension phenotype, as observed in the current study [37]. Overweight and obesity are considered preventable and treatable risk factors that have a similar effect as preeclampsia on the progression to cardiovascular and metabolic complications later in life.

Isolated diastolic hypertension (IDH) has consistently been associated with adverse cardiovascular events, particularly in young individuals, where it is more prevalent. Therefore, treatment is recommended for individuals under the age of 50 with IDH, as it has been shown to significantly reduce cardiovascular complications. It is worth noting that IDH in elderly subjects is less prevalent and is usually not associated with cardiovascular complications [38,39,40]. In a study involving over 10,000 women from the Finnish population with long-term follow-up (average of over 39 years), an increased risk of ischemic heart disease ranging from 5% to 50% was observed among those with IDH [41].

Interestingly, our results differ from those reported in the literature since the model showed pregestational BMI and GWG did not influence the development of arterial hypertension in the first year post-childbirth; many authors point out that maternal overweight and obesity and excess gestational weight gain influence the subsequent development of cardiovascular and metabolic diseases postpartum [42,43]. Although the crude analysis of our cases showed that 81 of 120 women (67.5%) with pregestational BMI ≥ 25 had preeclampsia (OR 2.30, 95%CI 1.38–3.80), this confirms that this is one of the risk factors associated with the complication. In the general linear model, this factor of pre-pregnancy overweight and obesity had no effect on blood pressure during the year of follow-up with our patients. The same thing happened with GWG.

There is documented evidence suggesting that if PE occurs at term and with a child with adequate birth weight, cardiovascular risk is doubled later in life, but if the pregnancy is full-term with an SGA newborn, the CV risk is tripled. When the birth is preterm, this risk increases to 5, and if preeclampsia occurs before week 34 of gestation, the CV risk will be tenfold times higher [44]. Nevertheless, the results of this study did not agree with these assumptions. No differences were found in the presentation of hypertension or metabolic syndrome between women with preterm and full-term births, deliveries before or after the 34th week of gestation, or among SGA, appropriate-for-gestational-age (AGA), or large-for-gestational-age (LGA) deliveries in any of the three evaluations. Increasing the sample size might help clarify these discrepancies in the results.

### 4.6. Hypertension and Insulin Resistance

The association between insulin resistance (IR) and blood pressure has been extensively studied in patients with diabetes mellitus. It is believed that RI plays a significant role in the development of hypertension. Proposed mechanisms to explain this include enhanced tissue angiotensin II activity and aldosterone, insulin oxidative stress, and increased sympathetic nervous system activity [45]. In the Women’s Health Study published in 2007, a direct and independent association between blood pressure and the incidence of diabetes mellitus was reported [46]. Subsequent studies have confirmed this relationship [47,48]. However, the results of the current (our) study regarding the association of IR with blood pressure were inconsistent. Insulin resistance (2.7 HOMA cut-off point) was significantly associated with systolic and diastolic hypertension at three months postpartum and higher mean blood pressure figures of 6.5 and 5.6 mm Hg, respectively. At six months postpartum, there were no statistical associations. Finally, at 12 months, only diastolic blood pressure was found to be associated with hypertension and insulin resistance, with a mean pressure of 4.8 mm Hg higher for the latter. The most important result on this topic is that insulin resistance (HOMA) was a factor with a main effect on the figures of blood pressure (systolic and diastolic) in the applied statistical model.

### 4.7. Metabolic Syndrome

Using the ATP III criteria for MS diagnosis, a high risk of developing MS during the first year postpartum was observed in women who had experienced preeclampsia.

Metabolic syndrome is considered a potential complication following preeclampsia. In a systematic analysis conducted by Udenze, the prevalence of post-preeclampsia MS ranged from 10.9% to 27.3%, with a reported risk for its development ranging from 1.23 to 3.60 [49]. The results of our work were inconclusive regarding the development of MS during the first year of postpartum, with odds ratios at six months not reaching significance. However, at three months, the odds ratio was 5.2, and at 12 months, it was 8.2, more than double the values reported by Udenze. The variability of MS was not solely explained by preeclampsia but also by pre-pregnancy obesity, which added weight and credibility to the results (R^2^ values ranging from 0.410 to 0.247). Similar to our study, Cho et al. also demonstrated an increased risk of developing metabolic syndrome postpartum in women without pre-pregnancy metabolic syndrome, with BMI showing a direct association with its development [12].

Another study reported a three-fold increased risk of metabolic syndrome associated with preeclampsia and gestational hypertension. Metabolic syndrome is a basic component of diabetes mellitus. Almost 10 years ago, a retrospective, population-based cohort study found that the risk of developing diabetes mellitus doubled after 16.5 years of pregnancy follow-up with pre-eclampsia or gestational hypertension, in the absence of prior diabetes [11]. Previously, a 3- to 4.4-fold increased risk of developing type 2 diabetes mellitus was found after pregnancy with both mild and severe preeclampsia [30].

In international literature, there are several studies that demonstrate the protective influence of exclusive breastfeeding in preventing the onset of metabolic syndrome and diabetes mellitus, which seems to be more effective at younger ages. The beneficial effect of breastfeeding has even been observed in women with gestational diabetes [50,51].

Our study on the potential impact of exclusive breastfeeding on the development of metabolic syndrome (MS) yielded conflicting results. While the 3-month evaluation found an unexpected positive correlation between metabolic syndrome and breastfeeding, we faced a limitation in the low number of women who exclusively breastfed their children for 6 and 12 months, which prevented us from obtaining sufficient statistical power for drawing reliable conclusions.

### 4.8. Maternal Adiposity

In this study, various measurements of adiposity, including BMI, waist-hip ratio, and waist-height ratio, were calculated during each assessment in the postpartum period. The results indicate that pre-pregnancy BMI explained a significant proportion (greater than 0.60) of the variability in BMI at 3, 6, and 12 months postpartum. On the other hand, gestational weight gain (GWG) accounted for a smaller proportion (between 0.10 and 0.25) of the variability, and preeclampsia (PE) explained between 0% and 2% of the model’s variability. This suggests that, in this particular study, PE did not significantly influence the development of postpartum overweight and obesity. However, both overweight/obesity and PE were strongly associated with high blood pressure and metabolic syndrome up to one year after pregnancy. These findings align with international literature, including a meta-analysis of prospective cohort studies that found an increased risk of preeclampsia in overweight or obese nulliparous and multiparous pregnant women, highlighting maternal adiposity as an independent risk factor for PE [52,53].

In a 2020 report by Lopes et al., it was observed that women with hypertensive disorders during pregnancy, particularly those with a pre-pregnancy BMI ≥ 35, experienced a slower resolution of their arterial hypertension postpartum [54]. Similarly, Miller et al. found that women with preeclampsia had higher mean postpartum blood pressure if they had a higher pre-pregnancy BMI, compared to women matched for BMI but without preeclampsia [55]. These findings are consistent with the results of the current study, further supporting the association between pre-pregnancy overweight/obesity, preeclampsia, and postpartum blood pressure levels. Overall, the evidence suggests that pre-pregnancy BMI plays a substantial role in postpartum adiposity and blood pressure outcomes, while gestational weight gain and preeclampsia have smaller contributions in this regard. The association between overweight/obesity, preeclampsia, and adverse postpartum cardiovascular outcomes underscores the importance of addressing these risk factors to mitigate long-term health consequences.

## 5. Conclusions

The study confirmed that women with a history of preeclampsia have an increased risk of developing arterial hypertension, particularly isolated diastolic hypertension, and metabolic syndrome. Likewise, with a similar or greater risk, overweight and obesity were associated with high blood pressure and metabolic syndrome in the first year postpartum. Furthermore, there was a strong and direct association between overweight/obesity and pre-pregnancy BMI, with pre-pregnancy obesity significantly influencing the development of metabolic syndrome.

This study found that being overweight or obese may have a similar effect as preeclampsia on the progression to cardiovascular and metabolic complications. Although there are still no ways to prevent or treat preeclampsia, we believe that overweight and obesity are preventable and treatable risk factors, and control of these would help reduce progression to MS and hypertension.

### Limitations

The follow-up of our patients was limited to three evaluations, which covered 12 months after the obstetric event. This could be considered a short follow-up since in the literature there are some reports that at a 2-year follow-up, MS has been found in 25% of women with a history of PE [18]. In this study, the time onset of PE was not considered, leaving for future studies to find out whether the early or late onset of PE plays a role in the subsequent development of hypertension and MS. We had a 25% loss of patients throughout a year of follow-up. However, we count in our favor that these losses were not differential from the group that completed the follow-up.

## Figures and Tables

**Table 1:** Post-childbirth evaluations [n (%)].

	3 Months	6 Months	12 Months
Preeclampsia, Severe	52 (19.9)	49 (22)	42 (21.6)
Preeclampsia, Mild	96 (36.8)	80 (36)	68 (35.1)
Controls	113 (43.3)	94 (42)	84 (43.3)
Total	261(100)	223 (100)	194 (100)

**Table 2:** Clinical characteristics of the initial study population [*n* (%)].

	Severe	Mild	Controls	Total	Sig.
	Preeclampsia	Preeclampsia			
	*n* = 52	*n* = 96	*n* = 113		
Age groups (years)					
18 to 25	16 (25.4)	21(33.3)	26 (41.3)	63	0.537
26 to 35	25 (16.8)	55 (36.9)	69 (46.3)	149	
36 to 45	11 (22.4)	20 (40.8)	18 (36.7)	49	
Pregestational BMI [kg/m^2^]					
18.4 or less	3 (15.8)	4 (21.1)	12 (63.2)	19	0.001
18.5 to 24.9	29 (23.8)	31 (25.4)	62 (50.8)	122	
25 to 29.9	14 (17.5)	43 (53.8)	23 (28.7)	80	
30 or more	6 (15.0)	18 (45.0)	16 (40.0)	40	
Personal history of					
Diabetes Mellitus					
Yes	0 (0)	2 (100)	0 (0)	2	0.117
No	52 (20.1)	94 (36.3)	113 (43.6)	259	
Personal history of					
High Blood Pressure					
Yes	9 (25.0)	24 (66.7)	3 (8.3)	36	<0.001
No	43 (19.1)	72 (32.0)	110 (48.9)	225	
Personal history of					
Gestational Diabetes					
Yes	3 (17.6)	8 (47.1)	6(35.3)	17	0.658
No	49 (20.1)	88 (36.1)	107 (43.9)	244	
Premature birth					
(<37 weeks gestational age)					
Yes	23 (37.1)	21 (33.9)	18 (29.0)	62	0.001
No	29 (14.6)	75 (37.7)	95 (47.7)	199	
Neonatal Birth Weight					
≤2500 g					
Yes	30 (48.8)	24 (38.7)	8 (12.9)	62	<0.001
No	22 (11.1)	72 (38.7)	105 (52.8)	199	
Weight for Gestational Age					
SGA	19 (40.4)	16 (34.0)	12 (25.5)	47	<0.001
AGA	25 (14.2)	71 (40.3)	80 (45.5)	176	
LGA	8 (21.2)	9 (23.7)	21 (55.3)	38	
Number of Pregnancies					
One	23 (31.1)	28 (37.8)	23 (31.1)	74	0.004
Two or three	25 (20.3)	42 (34.1)	56 (45.5)	123	
Four or more	4 (6.3)	26 (40.6)	34 (53.1)	64	
Personal history of					
previous preeclampsia					
Yes	15 (21.7)	45 (65.2)	9 (13.0)	69	<0.001
No	37 (19.3)	51 (26.6)	104 (54.2)	192	

**Table 3:** Arterial hypertension post-delivery [n (%)].

Months Post-Delivery	Preeclampsia Group	Control Group	Sig.	OR (95% CI)
Three				
Systolic BP				
≥140	7 (87.5)	1 (12.5)	0.740	5.56 (0.67, 45.8)
≤139	141 (55.7)	112 (44.3)		
Diastolic BP				
≥90	19 (90.5)	2 (9.5)	0.001	8.17 (1.86, 35.8) **
≤89	129 (53.8)	111 (46.3)		
Six				
Systolic BP				
≥140	5 (83.3)	1 (16.7)	0.190	3.79 (0.43, 32.9)
≤139	132 (56.9)	100 (43.1)		
Diastolic BP				
≥90	11 (78.6)	3 (21.4)	0.100	2.82 (0.76, 10.4)
≤89	118 (56.5)	91 (43.5)		
Twelve				
Systolic BP				
≥140	3 (100)	0 (0.0)	0.120 *	1.79 (1.58, 2.02) **
≤139	115 (55.8)	91 (43.5)		
Diastolic BP				
≥90	9 (100)	0(0.0)	0.007 *	1.83 (1.60, 2.08) **
≤89	101 (54.6)	84 (43.3)		

Hypertension systolic ≥ 140 mm Hg; Hypertension diastolic ≥ 90 mm Hg; * Fisher exact test; ** Relative risk of hypertension among those with a history of preeclampsia.

**Table 4:** Factors associated with the development of blood pressure figures in the postpartum.

	Systolic Arterial Pressure				Diastolic Arterial Pressure			
Months Post-Childbirth	B (95% CI)	Sig.	Eta^2^	R^2^	B (95% CI)	Sig.	Eta^2^	R^2^
Three								
Age	0.27 (0.05, 0.48)	0.014	0.024		0.29 (0.12, 0.46)	0.001	0.044	
Pre-BMI	−0.32 (−0.85, 0.21)	0.244	0.005		−0.20 (−0.64, 0.23)	0.357	0.003	
GWG	−0.22 (−0.46, 0.02)	0.071	0.013		−0.20 (−0.37, 0.01)	0.071	0.013	
Gestational Age	0.13 (−0.44, 0.71)	0.65	0.001		−0.11 (−0.58, 0.35)	0.63	0.001	
BMI	0.76 (0.21, 1.32)	0.007	0.029	0.210	0.80 (0.36, 1.25)	<0.001	0.048	0.262
HOMA	1.20 (0.43, 1.97)	0.002	0.037		0.70 (0.08, 1.32)	0.027	0.02	
Severe Preeclampsia	5.94 (1.89, 9.99)	0.004	0.033		3.45 (0.18, 6.71)	0.038	0.017	
Mild Preeclampsia	5.76 (2.29, 9.24)	0.001	0.041		2.89 (0.91, 5.68)	0.043	0.016	
History of previous preeclampsia	1.60 (−3.27, 8.47)	0.647	0.001		2.48 (−3.0, 8.02)	0.378	0.003	
Six								
Age	0.03 (−0.20, 0.28)	0.766	0		0.25 (0.64, 0.43)	0.009	0.033	
Pre-BMI	−0.39 (−0.99, 0.20)	0.189	0.008		−0.12 (−0.58, 0.32)	0.577	0.001	
GWG	−0.11 (−0.36, 0.14)	0.397	0.003		−0.15 (−0.34, 0.04)	0.122	0.011	
Gestational Age	−0.24 (−0.93, 0.41)	0.467	0.003		−0.02 (−0.53, 0.47)	0.909	0	
BMI	0.56 (−0.07, 2.09)	0.035	0.021	0.127	0.51 (0.03, 0.99)	0.036	0.021	0.166
HOMA	1.08 (0.76, 2.02)	0.039	0.02		0.54 (−0.22, 1.31)	0.166	0.009	
Severe Preeclampsia	3.66 (−0.79, 8.12)	0.107	0.012		1.13 (−2.26, 4.53)	0.511	0	
Mild Preeclampsia	4.91 (0.29, 8.90)	0.016	0.027		3.68 (0.64, 6.72)	0.018	0.027	
History of previous preeclampsia	2.12 (−5.20, 9.45)	0.568	0.002		2.29 (−3.29, 7.88)	0.419	0.003	
Twelve								
Age	0.32 (0.06, 0.57)	0.014	0.033		0.32 (0.13, 0.52)	0.001	0.058	
Pre-BMI	−0.40 (−0.97, 0.16)	0.164	0.011		−0.20 (−0.64, 0.23)	0.353	0.005	
GWG	−0.13 (−0.33, 0.06)	0.183	0.01		−0.12 (−0.31, 0.06)	0.205	0.009	
Gestational Age	0.03 (−0.62, 0.69)	0.912	0		0.27 (−0.23, 0.77)	0.291	0.006	
BMI	0.95 (0.36, 1.53)	0.002	0.053	0.258	0.77 (0.32, 1.22)	0.001	0.06	0.276
HOMA	0.16 (−0.06, 0.39)	0.161	0.011		0.26 (0.80, 0.44)	0.004	0.046	
Severe Preeclampsia	9.34 (4.66, 14.03)	<0.001	0.079		5.61 (2.03, 9.19)	0.002	0.05	
Mild Preeclampsia	7.67 (3.50, 11.85)	<0.001	0.068		4.15 (0.95, 7.34)	0.011	0.035	
History of previous preeclampsia	0.33 (−7.46, 6.79)	0.926	0		0.74 (−4.70, 6.20)	0.787	0	

Univariate general lineal model; GWG = Gestational weight gain. Pre-BMI = Pregestational body mass index. HOMA = Homeostatic model assessment.

**Table 5:** Factors associated with the development of metabolic syndrome after a pregnancy with preeclampsia.

Months Post-Childbirth	B (95% CI)	Sig.	R^2^
Three			
Age	0.94 (0.88, 1.02)	0.184	0.418
Pre-BMI ≥ 30	0.24 (0.81, 0.71)	0.014	
Pre-BMI 25–29.9	0.07 (0.21, 0.24)	<0.001	
Exclusive breastfeeding	0.76 (0.20, 0.29)	<0.001	
History of Gestational Diabetes	3.98 (0.50, 31.3)	0.189	
GWG	1.03 (0.96, 1.12)	0.352	
Severe Preeclampsia	0.28 (0.06, 1.17)	0.081	
Mild Preeclampsia	0.19 (0.05, 0.65)	0.009	
LDL-C	0.57 (0.21, 1.56)	0.236	
History of previous preeclampsia	0.52 (0.20, 1.36)	0.185	
Six			
Age	1.02 (0.94, 1.11)	0.597	0.298
Pre-BMI ≥ 30	0.12 (0.02, 0.64)	0.013	
Pre-BMI 25–29.9	0.02 (0.005, 0.13)	<0.001	
Exclusive breastfeeding	1.06 (0.37, 3.05)	0.906	
History of Gestational Diabetes	0.43 (0.08, 2.11)	0.299	
GWG	1.02 (0.93, 1.11)	0.619	
Severe Preeclampsia	0.59 (0.15, 2.30)	0.454	
Mild Preeclampsia	0.78 (0.22, 2.76)	0.705	
LDL-C	0.72 (0.23, 2.25)	0.58	
History of previous preeclampsia	0.91 (0.29, 2.80)	0.871	
Twelve			
Age	0.99 (0.87, 1.12)	0.931	0.272
Pre-BMI ≥ 30	0.22 (0.36, 1.44)	0.116	
Pre-BMI 25–29.9	0.1 (0.15, 0.75)	0.021	
Exclusive breastfeeding	0.53 (0.05, 5.37)	0.597	
History of Gestational Diabetes	0.33 (0.04, 2.64)	0.299	
GWG	0.98 (0.87, 1.11)	0.783	
Severe Preeclampsia	0.1 (0.01, 1.08)	0.058	
Mild Preeclampsia	0.33 (0.03, 3.43)	0.355	
LDL-C	0.32 (0.06, 1.76)	0.196	
History of previous preeclampsia	0.5 (0.12, 2.01)	0.329	

(new ATP III criteria) [27]. Sig: significance.

**Table 6:** Role of preeclampsia and other factors in the development of adiposity after delivery.

Months After-Childbirth	B (95% CI)	Eta^2^	Sig.	R^2^ Adj.
Three				
Body Mass Index				
Pre-BMI	0.833 (0.765, 0.900)	0.706	<0.001	0.725
GWG	0.209 (0.161, 0.256)	0.256	<0.001	
Preeclampsia	0.367 (−0.368, 1.102)	0.004	0.326	
Waist Hip Index				
Age (years)	0.001 (0.000, 0.002)	0.032	0.005	0.13
Pre-BMI	0.003 (0.001, 0.004)	0.058	<0.001	
GWG	−0.001 (−0.002, −0.000)	0.020	0.026	
Preeclampsia	0.019 (−0.058, 0.095)	0.020	0.631	
Height Waist Index				
Pre-BMI	1.002 (0.893, 1.111)	0.572	<0.001	0.595
GWG	0.158 (0.081, 0.235)	0.063	<0.001	
Preeclampsia	−0.583 (−6.714, 5.548)	0.000	0.852	
Six				
Body Mass Index				
Pre-BMI	0.831 (0.755, 0.907)	0.692	<0.001	0.711
GWG	0.157 (0.106, 0.209)	0.151	<0.00	
Preeclampsia	2.159 (−1.145, 5.463)	0.008	0.199	
Waist Hip Index				
Age (years)	0.001 (0.000, 0.002)	0.027	0.018	
Pre-BMI	0.003 (0.001, 0.004)	0.065	0.001	0.19
Preeclampsia	−0.024 (−0.081, 0.034)	0.003	0.419	
Height Waist Index				
Pre-BMI	0.991 (0.874, 1.109)	0.572	<0.001	
GWG	0.101 (0.022–0.180)	0.030	0.012	0.612
Preeclampsia	−1.531 (−6.642, 3.580)	0.002	0.555	
Twelve				
Body Mass Index				
Pre-BMI	0.775 (0.689, 0.861)	0.636	<0.001	0.646
GWG	0.134 (0.074, 0.194)	0.097	<0.001	
Preeclampsia	−2.589 (−6.540, 1.362)	0.009	0.198	
Waist Hip Index				
Age (years)	0.002 (0.001, 0.003)	0.043	0.005	0.155
Pre-BMI	0.002 (0.001, 0.004)	0.049	0.003	
Exclusive breastfeeding	0.043 (0.012, 0.073)	0.040	0.007	
Preeclampsia	−0.004 (−0.075, 0.068)	0.000	0.922	
Height Waist Index				
Pre-BMI	0.956 (0.816, 1.096)	0.501	<0.001	0.525
GWG	0.105 (0.007, 0.202)	0.024	0.035	
Exclusive breastfeeding	3.357 (0.579, 6.136)	0.030	0.018	
Preeclampsia	−2.808 (−9.235, 3.619)	0.004	0.390	

Univariate linear general model. Adjusted by age, breastfeeding practice, use of hormonal contraceptives, gestational weight gain, pre-pregnancy BMI, and having or not having preeclampsia (results included in the table). Pre-BMI: pregestational (WHO); GWG: gestational weight gain; Adj: adjusted; Underline：the different outcome variables (adiposity).

## Data Availability

The data for this study will not be available for now because they are part of a multidisciplinary study that is still in progress.
